# Evaluation of neurological changes in secondary progressive multiple sclerosis patients treated with immune modulator MIS416: results from a feasibility study

**DOI:** 10.1186/s40814-017-0201-4

**Published:** 2017-11-16

**Authors:** Gill A. Webster, Dalice A. Sim, Anne C. La Flamme, Nancy E. Mayo

**Affiliations:** 1 Innate Immunotherapeutics Ltd, Auckland, New Zealand; 2 University of Otago, Wellington, New Zealand; 3 Victoria University, Wellington, New Zealand; 4 McGill University, Montreal, Canada

**Keywords:** Secondary progressive multiple sclerosis, MIS416, Immune modulator, Myeloid cells, Neurological improvement, Performance related outcome, Patient-reported outcome, Plasma immune biomarker

## Abstract

**Background:**

While disease progression can be readily monitored in early stage relapsing multiple sclerosis (MS), it is more challenging for secondary progressive multiple sclerosis (SPMS). This advanced stage of disease has distinct pathophysiology due to compartmentalization of neuroinflammatory activity within the central nervous system, resulting in increased incidence and severity of cognitive dysfunction. The shift in the dominant disease pathways is underscored by the failure of relapsing therapies to benefit SPMS patients, highlighting the need for novel treatment strategies and clinical trial endpoints that are well-aligned with potential benefits. The Expanded Disability Status Scale (EDSS) is widely used but is weighted towards ambulatory ability, lacking sensitivity to other aspects of neurological impairment experienced in more severely disabled SPMS patients, so may not effectively capture their clinical status.

To investigate the feasibility of an alternative clinical trial endpoint model for a phase 2B trial of an immune modulator for SPMS, the potential for treatment efficacy-based patient-centered outcomes was assessed within the context of a before and after, 12-week clinical trial of safety and tolerability.

**Methods:**

Patients treated with MIS416 for 12 weeks were evaluated for clinical status at baseline and end of dosing, using the established Multiple Sclerosis Functional Composite, Short Form Health Survey, and Expanded Disability Status Scale. Responder status was determined for eight outcome measures based on minimally important change, defined using published studies. To evaluate the patients’ immune response to MIS416, blood plasma samples collected at baseline and pre- and 24-h post doses 1–4 were analyzed using multiplex cytokine quantification assays.

**Results:**

Using a combination of patient-centered outcomes, MIS416 treatment was associated with improved clinical status for 10/11 patients: eight patients showed improvement on two to five outcome measures, five of which also showed improvement by EDSS. Multi-dimensional scaling analysis of MIS416-induced factors quantified in individual patients, revealed immune response patterns which had a strong concordance with the extent of the patients’ clinical response.

**Conclusions:**

The data support the feasibility of using patient-centered outcomes as additional clinical trial endpoints, for determining the efficacy of disease-modifying therapies, in secondary progressive multiple sclerosis patients.

**Trial registration:**

ClinicalTrial.gov, NCT01191996

## Background

Multiple sclerosis (MS) is a chronic autoimmune disease of the central nervous system (CNS) [1], resulting in the occurrence of inflammatory lesions (plaques) anywhere within the white matter of the CNS, most commonly the periventricular regions, optic nerves, brain stem, cerebellum, and spinal cord [1, 2]. The symptoms and impairments experienced by people with MS vary and depend on the affected areas of the CNS [3, 4]. MS affects three times as many women as men and is typically diagnosed between the ages of 15 and 40 years [5]. It is estimated that there are 2.3 million individuals with MS worldwide [6]. The highest prevalence of MS is found in Canada, USA, and other northern European countries with rates ranging from 50 to 240 per 100,000 population [6, 7]. The most common type of MS is relapse-remitting MS (RRMS) [8], affecting 75–85% of patients, and characterized by clear and well-defined relapses followed by complete or partial remissions [8]. About 50% of individuals diagnosed with RRMS will develop secondary progressive MS (SPMS) after 10–15 years [8]. SPMS is characterized by steady worsening of the condition, with or without attacks and remissions [8]. Primary progressive MS (PPMS) affects about 10% and is characterized by continuous and slow worsening of symptoms from the onset of the condition, with no clear relapses or remissions [8]. The least common and most severe type of MS is progressive-relapsing MS (PRMS), which occurs in about 5% of the persons diagnosed with MS [8]. This type is characterized by steady worsening of the condition with clear attacks and without remissions [8].

MIS416 is a myeloid-targeted immune response modifier currently in a phase 2B randomized, double-blind, placebo-controlled trial to study the efficacy and safety in the treatment of subjects with secondary progressive multiple sclerosis (SPMS) (http://clinicalTrials.gov identifier NCT02228213). The data from a clinical trial to determine safety and tolerability has been previously published [9]. In contrast to relapse remitting MS (RRMS) therapies that target the peripheral “outside-in” adaptive immune biology, MIS416 has been developed to modulate the secondary progressive stage of MS, which is considered to reflect self-perpetuating innate inflammation that has become contained within the central nervous system (CNS) [10]. This shift in disease pathophysiology is emphasized by the failure of RRMS therapies to alter disease progression in SPMS patients [11]. Early pre-clinical and human compassionate use studies have demonstrated the ability of MIS416 to enhance several myeloid-directed anti-inflammatory pathways that can potentially access the CNS, leading to inhibition of this compartmentalized neuroinflammation and the promotion of endogenous CNS repair pathways [12, 13]. Accordingly, to fully evaluate the clinical potential of MIS416, measures of CNS function that are sensitive to SPMS-associated chronic neuroinflammatory status are desirable.

Currently, the Expanded Disability Status Scale (EDSS) is the only regulatory-approved measure of change in disease activity and burden in MS patients [14]. However, reported limitations of the EDSS [14], such as lack of sensitivity to cognitive and arm function, mean that for patients with advanced disease, improvements in clinical status may not be captured by EDSS, which is biased towards the patient’s walking ability [15]. Given the recognized limitations of the EDSS, there is increased focus on the refinement and validation of patient-reported outcomes (PROs) and performance-related outcomes (PerfOs), to account for the full range of MS symptoms [16, 17]. This shifting ground with respect to the important outcomes of MS therapy and the need to include patient-centered outcomes [18] raises uncertainty about the feasibility of including outcomes beyond the EDSS in trials to determine the treatment effect of MIS416 in SPMS patients. A recent paper by the Multiple Sclerosis Outcome Assessments Consortium (MSOAC) [19] has outlined the need for better outcome measures for MS. In preparation for a shift in the recommended outcomes for MS, this study aimed to investigate the feasibility of an alternative clinical trial endpoint model. Accordingly, we conducted a preliminary analysis of PRO and PerfO secondary endpoint measures from the dose-confirmation phase of a trial on MIS416-treated SPMS patients, to provide data for assessment of response on the patient-centered outcomes. As peripheral blood immune biomarkers associated with MIS416 mechanism of action are known [9, 20], patient samples collected immediately prior to and 24 h after MIS416 administration, for doses 1–4, were analyzed to determine any relationship between the pattern of the patient’s immune response to MIS416 and their change in clinical status, as indicated by these PerfOs and PROs. The overall aim was to support the feasibility of using patient-centered outcomes to measure response to MIS416 treatment in SPMS patients in a larger, placebo-controlled efficacy study.

## Methods

### Clinical trial design

A single-center, open-label, non-randomized, dose-escalation study was conducted in two phases: a dose-escalation (DE) phase, to evaluate the safety, tolerability, and maximum tolerated dose (MTD) of MIS416 administered intravenously, once weekly for four doses; and a dose-confirmation (DC) phase, comprising a single cohort treated at or below the MTD of MIS416, dosed once weekly for up to 12 doses [9]. The study was conducted in accordance with the Declaration of Helsinki [21] and was approved by the Upper South A Health and Disability Ethics Committee (URA/10/01/011). Written informed consent for blood sample collection and analysis was obtained from all study participants prior to the trial. The methods we are reporting follow the recommendations from the new CONSORT extension to randomized pilot and feasibility trials [22].

### Patients

Study participants were recruited from Christchurch, New Zealand, where the clinical trial site was located. All patients provided informed consent prior to screening. Exclusion criteria included treatment with any immunomodulatory therapy in the previous 6 months or vaccine/corticosteroid in previous 60 days, as well as any diseases that might impact on the patients’ diagnosis and evaluation of MS. Altogether, 34 patients (20 females) 18 years or older were enrolled, 19 in the DE and 15 in the DC phase. All had a diagnosis of MS based on McDonald’s criteria [23], either primary or secondary progressive MS, evidence of worsening clinical status over the previous 2 years, and EDSS scores of 2.5–7.0 at screening. Enrollment in the DC phase was limited to patients with SPMS, to support a planned phase 2 trial in this more homogeneous population. The feasibility study to investigate patient-centered outcomes was conducted on the DC cohort only.

### Patient clinical status and measures

The clinical status of patients who completed the DC phase (MIS416 weekly for 12 weeks) was assessed before and after completion of the study using the EDSS, Multiple Sclerosis Functional Composite (MSFC) [24], Fatigue Severity Scale (FSS) [25], and the Short Form Health Survey-36 (SF-36) [26].

### Calculation of change and responder status on performance-rated outcome measures and patient-reported outcome measures (patient-centered outcomes)

Minimal important change (MIC) was used to define a positive patient responder status as estimated from published studies. For EDSS, MIC was 0.5 [27]. From the MFSC [24], MIC values were determined for the following performance-rated outcomes (PerfOs): gait speed (GS) 0.10 m/s [28]; Paced Auditory Serial Addition Test (PASAT) 9 (based on changes greater than estimates of the practice effect) [29, 30]; Nine-Hole Peg Test (NHPT) 20% [29]. For the SF-36, four subscales that were most closely related to the biological action of the investigational drug were included as outcomes. These were physical function to reflect the everyday impact of improved gait speed or walking; mental health to reflect both a primary effect or a secondary effect from walking better; vitality for fatigue as it is the most distressing symptom of people with MS and not at all captured by the EDSS; and general health as improved walking, mood, and fatigue will impact this outcome and it is an important patient-centered outcome [31]. The other four subscales, pain and the three role subscales, were not included, as pain is often either neuropathic in origin or musculo-skeletal secondary to abnormal walking pattern, and the role variables are downstream outcomes from improved mobility and mental health.

For these subscales, a change in 10 points on a 0–100 scale for each score was considered a MIC [32] (equivalent to 1/2 standard deviation (SD) as SD is approximately 20 in adults > 55 years from a large nominative sample) [30]. The FSS was originally included in the test battery, but we did not want to include two PROs which both measured fatigue, and as the other PROs came from the SF-36, to add homogeneity, we included only SF-36 PROs. In addition, the items of the FSS (*n* = 9) do not measure severity of fatigue but rather causes, consequences, and impact on daily life.

Each patient was classified as a responder (1) or non-responder (0) on the EDSS and the three PerfOs and four PROs from the SF-36. Response across all measures was summarized using the total number of measures with an observed response. Response pattern was used to rank patients based on total number of responses with priority given to responses on PerfO. Patients were then classified into three groups based on the distribution of ranks: high, medium, and low responder.

### Quantification of plasma MIS416 immune biomarkers analysis

For quantification of immune factors in patient plasma, heparin anti-coagulated peripheral blood was collected pre-treatment (baseline), as well as pre- and 24-h post MIS416 administration for doses 1–4. Following immediate processing of blood, plasma was isolated and stored at − 80 °C until analysis. The immune factors selected were based on their capacity to reflect different aspects of MIS416-mediated immune activation, as determined in a pilot study conducted on plasma from patients who completed the DE phase of the trial: type I/II interferon signaling (IP-10, MIG, MIP-1α, neopterin, IFN-γ); pro-inflammatory mediators (IL-6, IL-11, GCSF, IL-12p40, PGE2); cell migration (fractalkine, rantes, MCP-1); anti-inflammatory mediators (TGF-β, soluble TNFR1, IL-10, IL-1RA); cell adhesion (CD62E, ICAM1, VCAM1); and the growth factor, VEGF. The concentrations (pg/mL) of these cytokines and chemokines were determined using a custom cytokine bead array matrix (Becton Dickinson CBA Flex Sets™) or by ELISA (BD Biosciences) according to the manufacturer’s instructions.

### Statistical analysis

#### Patient-centered outcomes

Each patient was classified as a responder or non-responder on the EDSS and the three PerfOs and four PROs from the SF-36. Response across all measures was summarized using the total number of measures with an observed response. Participants were ranked in order of total responses across PerfOs and PROs. The probability of observing response patterns of the magnitude determined was estimated by referring to the binomial distribution and assuming a spontaneous response rate to be rare (≤ 3/10), given the population under study.

#### Immunological factors

To evaluate the extent to which each patient’s immune system was altered in response to MIS416 therapy, the maximum recorded plasma concentration for each immune factor across dose 1–4 time points was used for further analysis. The data were first normalized (subtracted the mean and divided by the standard deviation) so that all were equally weighted, then the resemblance between each pair of patients was determined. This was calculated as the sum (over all immune factors) of the squared differences between patients. Thus, for each pair (i, j) of patients,

$$ \mathrm{Resemblance}\;\left(i,j\right)={\sum}_{21}{\left({V}_i-{V}_j\right)}^2 $$

where *V*_*i*_ and *V*_*j*_ are the values for patients *i* and *j* respectively. Once the resemblances were calculated, a cluster algorithm was used to generate a multi-dimensional scaling analysis plot to illustrate the differences between patients. SIMPER (similarity percentages) analysis was used to determine which immunological factors mostly accounted for the differences between patients [33, 34]. These factors were used subsequently to compare the patient immune responses with the patient-centered clinical response.

#### Statistical analysis of immune response biomarkers in patients grouped by clinical responder status

Biomarker levels for patients grouped according to their clinical responder status (high, medium, or low) were compared using a two-way ANOVA followed by Holm-Sidak’s multiple comparison post-test (PRISM software version 7.0; GraphPad). A value of *P* < 0.05 was taken as significant.

## Results

Of the targeted 15 patients enrolled in the DC phase, 11 subjects completed all 12 weekly doses. Two subjects withdrew for personal reasons and 2 subjects were withdrawn due to an adverse event which occurred early on in dosing schedule. Adverse events were headache (*n* = 2) and pain in extremity (in the arm or the leg) (*n* = 1).

The distribution of clinical status measures prior to MIS416 treatment of the patients who completed the DC phase [9] are summarized in Table 1.

**Table 1:** Distribution of the clinical characteristics of the patient sample group (DC cohort; *n* = 11) for age, EDSS, PerfOs, and PROs prior to MIS416 treatment

	Median	Minimum	Maximum
Age (years) (*n* = 11)	53	46	60
EDSS	6	4	7
^a^Gait speed (m/s)	0.68	0.26	1.19
NHPT (s)	28.5	19.8	152.3
^b^PASAT (errors)	37	23	60
^c^SF-36 [Norm]/100			
General health [75]	46.1	23.7	65.1
Physical function [82]	23.1	19.3	40.3
Bodily pain [75]	42.6	30.6	62
Role physical [81]	30.2	2.5	39.2
Role emotional [88]	45.7	17.9	56.2
Social function [88]	37.3	17.2	57.3
Vitality [68]	40.7	25.9	52.6
Mental health [80]	50.9	29.9	61.3

^a^ *n* = 10

^b^Maximum score possible = 60

^c^Higher = better health

### Patient change and responder status on EDSS, PerfOs, and PROs

The amount of change on the EDSS and the three PerfO measures in absolute terms, or percent change from baseline for the NHPT, as well as how each patient was classified on responder status for each of the measures is shown in Table 2.

**Table 2:** Change and responder status (RS) on EDSS and PerfOs

Patient ID	EDSS		Gait speed		PASAT		NHPT	
	Change	RS	Change	RS	Change	RS	% change	RS
DC01	3.5	*1*	0.10	*1*	9	1	− 3.48	0
DC02	0	0	− 0.05	0	− 14	0	1.91	0
DC03	− 0.5	0	− 0.17	0	− 2	0	− 4.81	0
DC05	1	*1*	0.10	*1*	0	0	1.40	0
DC06	0	0	0.035	0	1	0	− 7.30	0
DC07	0.5	*1*	0.14	*1*	6	0	− 5.03	0
DC09	0	0	0.07	0	15	1	− 1.44	0
DC10	0.5	*1*	0.14	*1*	− 4	0	− 10.26	0
DC11^a^	0	0	.	.	11	1	− 13.79	0
DC12	0.5	*1*	− 0.10	0	24	1	42.42	0
DC14	0	0	0.04	0	0	0	7.53	0

^a^Non-ambulatory patient hence no gait speed measures

Five patients were responders on EDSS and 4 of those also responded on gait speed. There were 4 responders on PASAT and 0 on NHPT. In all, 7/11 patients were responders on at least one of these measures.

Table 3 shows the amount of change in absolute terms for the four PROs. Of these, there were 6 responders on vitality; 3, 2, and 1 patients responded on general health, mental health, and physical functioning respectively, with a total of 8/11 patients responding on at least one PRO.

**Table 3:** Change and responder status (RS) on PROs

Patient ID	Physical function		General health		Vitality		Mental health	
	Change	RS	Change	RS	Change	RS	% change	RS
DC01	17.2	1	7.1	0	14.9	1	7.8	0
DC02	0	0	0	0	14.9	1	2.6	0
DC03	0	0	12.9	1	− 2.9	0	2.6	0
DC05	9.6	0	− 2.4	0	11.9	1	10.5	1
DC06	5.3	0	− 9.6	0	11.9	1	10.5	1
DC07	3.8	0	− 7.1	0	− 3	0	− 5.2	0
DC09	9.6	0	14.3	1	2.9	0	5.2	0
DC10	− 1.9	0	9.5	0	14.9	1	7.9	0
DC11	7.6	0	14.3	1	11.9	1	5.3	0
DC12	0	0	1.4	0	− 8.9	0	− 23.6	0
DC14	0	0	− 2.4	0	0	0	− 2.6	0

For EDSS/PerfOs the probability of observing 7/11 responders, even assuming a spontaneous response as high as 30% in the absence of intervention is 0.022. The probability of observing 8/11 responders on PRO is 0.004 (http://stattrek.com/online-calculator/binomial.aspx).

Table 4 shows how each patient was classified as a responder ranked in order by total number of responding outcomes. While the ranking of patients was based on total number of responses, priority was given to responses on PerfO. All but one patient (DC14) showed a response on at least one variable. The patient ranking was further classified as high, medium, and low responder based on ≥ 3, 2, and ≤ 1 total number of responses accordingly (Table 4).

**Table 4:** Number of responses on PerfOs and PROs, responder rank, and classification of responder status

Patient ID	PerfO	PRO	Total	Rank	Classification
DC01	3	2	5	1	High
DC05	2	2	4	2	High
DC10	2	1	3	3	High
DC11	1	2	3	4	High
DC12	2	0	2	5.5	Medium
DC07	2	0	2	5.5	Medium
DC09	1	1	2	7	Medium
DC06	0	2	2	8	Medium
DC02	0	1	1	9.5	Low
DC03	0	1	1	9.5	Low
DC14	0	0	0	11	Low

### Concordance of clinical responder status ranking with MIS416 pharmacodynamic immune response

MIS416 is composed of immune stimulatory ligands for innate receptors, toll-like receptor 9 (TLR-9), and nucleotide-binding oligomerization domain-containing protein 2 (NOD-2) [20]. As a result of immune crosstalk under the influence of TLR-9-dependant type I interferon signaling and NOD-2-dependant NFκB signaling, regulatory and anti-inflammatory immune activity can be established [12, 13, 35, 36]. Analysis of the patients’ immune response demonstrated that immune proteins associated with these pathways including the regulatory immune factors IFN-γ [37] and IL-10 [38] were transiently increased in patients’ plasma following MIS416 administration (maximal responses measured for each patient/immune factor are summarized in Table 5).

**Table 5:** Maximum value of immune factors measured in sequential peripheral blood plasma samples collected at 24-h and 7-day post doses 1, 2, 3, or 4

	Patient ID										
Immune factor (pg/mL)	DC	DC	DC	DC	DC	DC	DC	DC	DC	DC	DC
	1	2	3	5	6	7	9	10	11	12	14
CD62E	18,503	33,350	16,905	21,510	18,458	37,502	19,729	20,984	27,616	18,471	8447
Fractalkine	531	907	421	458	434	350	492	308	397	143	373
GCSF	28	24	29	25	26	30	24	27	25	21	26
ICAM1 x104	70	74	51	38	64	145	59	48	66	21	40
IFN-γ	178	281	147	74	73	192	76	48	52	0	122
IL-10	11	19	12	8	7	27	10	7	11	5	29
IL11	322	423	2540	3194	19	0	179	808	168	1522	60
IL12p40	339	321	182	291	292	185	60	183	65	136	177
IL1RA	6693	7696	5797	6315	3939	18,299	18,709	8070	6858	2721	4965
IL6	36	47	181	22	32	87	81	19	23	11	140
IP10	3640	5371	6089	2812	3068	7689	4240	1646	2704	1234	1395
MCP-1	163	722	889	432	330	296	385	187	255	704	465
MIG	9766	111,584	13,706	24,823	5444	18,058	16,966	7520	2780	1514	2989
MIP1-α	4	9	6	5	8	4	5	5	5	6	9
Neopterin	5237	3378	3486	2244	4221	3927	3850	3728	3891	2060	2658
PGE2	182	219	157	160	192	212	343	398	296	207	32
RANTES	255,145	174,684	189,866	231,938	166,300	257,508	353,761	229,399	265,178	167,513	119,622
TGF-β	6034	5973	3394	3872	4699	6542	6291	11,851	8124	8236	5047
TNF-R	4850	5230	5060	4560	7870	3680	2970	2450	1930	5210	2510
VCAM-1	972,534	761,399	970,295	1,047,583	1,300,968	889,864	672,495	1,056,576	467,492	936,152	828,607
VEGF	116	67	28	37	76	45	144	88	80	81	9

**Fig. 1:**
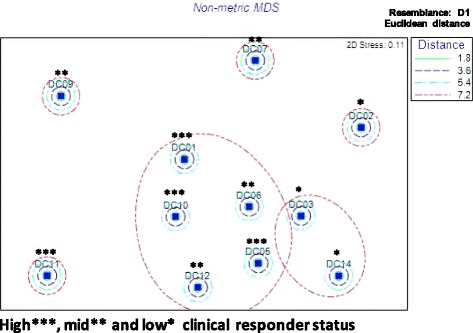
Multidimensional scaling DS plot illustrating similarities between patients based on their immune response to MIS416. The maximum level of immune factors detected in patient plasma following MIS416 treatment (Table 5) was used to compare each patient’s overall immune response to MIS416 with the group. Clusters of patients with similar immune responses are outlined as a group. The patient responder status based on clinical improvement as determined in this study (Table 4) is indicated

The multidimensional analysis of the maximal response (normalized) for each immunological parameter is presented as a non-metric multidimensional scaling plot (Fig. 1).

The clustering pattern for the 11 patients based on similarities of their immune response pattern to MIS416 showed a major cluster defined by 5 patients, and a small cluster of 2 patients, with 4 patients showing no clustering. This grouping of patients based on their immune response demonstrated a high degree of overlap with the grouping of patients based on their clinical response ranking. High clinical responders were defined by having demonstrated at least three responses on PerfO and PRO, medium responders had two responses, and low responders had 0–1 (Table 4). Out of the major immunological cluster comprising 5 patients, 3 of these were also the highest responders on patient-centered outcomes and the other 2 were medium responders. Of the small immunological cluster of 2 patients, both of these were the lowest responders on the patient-centered outcomes.

**Fig. 2:**
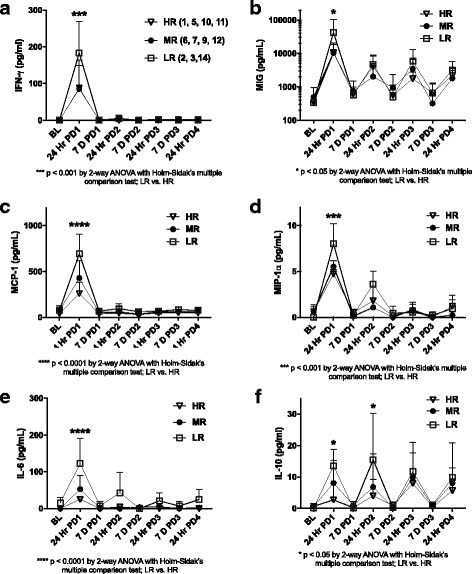
MIS416 pharmacodynamic immune response of patients grouped according to change in clinical status. Immune proteins most important for discriminating differential immune responses to MIS416: (IFN-γ (**a**), MIG (**b**), MCP-1 (**c**), MIP-1α (**d**), IL-6 (**e**), and IL-10 (**f**)) were quantified at 24-hour (24 Hr) and 7 days (7 D) post doses (PD) 1–4 and values were compared between the patient groups defined as high responder (HR), medium responder (MR) or low responder (LR) based on their extent of clinical response. The patient ID numbers comprising each responder group are shown in parenthesis. Data shown are the mean values (pg/mL) ±SD. Statistical significance was determined by two-way ANOVA followed by Holm-Sidak’s multiple comparison: *p< 0.05;**p< 0.005; ***p< 0.0001

Based on SIMPER analysis of the maximum observed value of each immune factor, those which provided the most information in discriminating patient immune responses to MIS416 were IFN-γ, MCP-1, MIG, MIP-1α, IL-6, and IL-10. To further examine the nature of these differences, the pattern of induction of immune factors by MIS416 by the high, medium, and low clinical responder groups were compared (Fig. 2).

The maximum levels of all immune factors, apart from IL-10, were detected at 24-h post-dose 1, and the responses were attenuated following subsequent doses. While this pharmacodynamic pattern of response was consistent for all patients, there were clear quantitative differences in the immune response to MIS416. Notably, low clinical responders had higher levels of IFN-γ and IFN-inducible proteins MCP-1, MIG, and MIP-1α than patients that were classified as high clinical responders. Furthermore, there were also higher levels of the NFκB-dependant cytokines IL-6 and IL-10 [38, 39] in patients that were classified as low responders. Patients classified as medium responders aligned more closely with either the high- or the low-responder groups or for IL-6 and IL-10, they were midway between the two.

## Discussion

### Clinical-patient-reported outcomes

Of the 11 patients assessed here, 5 were classified as responders on the EDSS and of these, 4 were classified concordantly on gait speed, 2 on the PASAT, with no responders determined based on the NHPT. On any one of these 4 PerfOs, 7 people would have been classified as responders with 2 people showing a response uniquely on PASAT. For the PROs, only 1 of the EDSS responders showed a response on physical function (SF-36 PF) and this was the one person with a large EDSS response (DC01: EDSS response + 3.5). This may indicate only large changes on the EDSS will translate to a meaningful improvement in function from the patient’s perspective. Other EDSS responders also showed response on vitality (*n* = 3) and mental health (*n* = 1). However, 5 people who were not EDSS responders showed response on one or more of the PROs. In an unblinded trial, PROs may change because patients wish to please the investigators and report more favorably on these outcomes. However, only four of the patients (DC02, DC03, DC06, DC11) did the response prevalence on PROs exceed that of the PerfOs suggesting that self-report bias may not have been responsible for the response pattern observed here. PROs are also affected by a phenomenon called response shift [18], in which patients recalibrate their responses over time based on a change in perspective. Again, the concordance between the PerfO and PRO response, or the more frequent PerfO response (*n* = 7), does not support recalibration as the sole mechanism affecting the PRO response, suggesting that true change occurred.

### Patient immune response

The observation that repeated dosing of MIS416 resulted in lower concentrations of biomarkers than were determined after dose 1 reflects desensitization of MIS416-stimulated immune pathways. Typically, this pattern of response is associated with repeat exposure to therapeutics or ligands that engage IFN and NFκB signaling and their respective negative feedback pathways [35, 40–42]. This intrinsic regulatory mechanism is geared to control the host inflammatory response and is central to the maintenance of immune and tissue homeostasis [43]. That MIS416 showed activation of negative feedback pathways in patients is further evidence that NOD-2 and TLR-9 pathway activation by MIS416 occurred within the limits of immune homeostasis, which is important from both a safety and therapeutic standpoint.

### Immune-clinical relationship—patient-reported outcomes

Analysis of the immunological response of the patients to MIS416 therapy supported their ranking based on the extent of their clinical improvement. While all patients responded immunologically in the same manner to MIS416, the patients that showed less clinical improvement had higher levels of MIS416 plasma biomarkers than those who were ranked as high responders. In particular, there were significantly more NFκB-dependant cytokines produced in these patients, although the significance (*p* value) of these differences should be treated with caution due to the small sample size. Such MIS416 hyper-responsiveness may be due to NFκB gene and pathway mutations described in MS patients which are associated with higher constitutive NFκB activity and greater sensitivity to NFκB-activating agents [44, 45].

## Conclusions

This study was limited by the low number of patients as well as the lack of a placebo control group. Notwithstanding these limitations, the results support the feasibility of pursuing these associations in a larger, placebo-controlled trial of MIS416 in SPMS patients and the evaluation of clinical status using a wider portfolio of patient-centered outcomes. Furthermore, in the context of such a study, concomitant analysis of the patient’s immune response to MIS416 may provide additional insight into the significance of any change in clinical status measures, in particular those which are sensitive to change in inflammatory activity within the CNS.

## Abbreviations

CD62E: E selectin
CNS: Central nervous system
DC: Dose confirmation
DE: Dose escalation
EDSS: Expanded Disability Status Scale
ELISA: Enzyme-linked immunosorbent assay
FSS: Fatigue Severity Scale
GCSF: Granulocyte colony-stimulating factor
ICAM1: Intracellular adhesion molecule 1
IFN-γ: Interferon gamma
IL: Interleukin
IL-1RA: Interleukin 1 receptor antagonist
IP-10: IFN-γ-inducible protein of 10 kDa
MCP-1: Monocyte chemotactic protein-1
MIC: Minimal important change
MIG: Monokine induced by γ-interferon
MIP-1α: Macrophage inflammatory protein
MSFC: Multiple Sclerosis Functional Composite
MTD: Maximum tolerated dose
NFκB: Nuclear factor kappaB
NHPT: Nine-Hole Peg Test
NOD-2: Nucleotide-binding oligomerization domain-containing protein 2
PASAT: Paced Auditory Serial Addition Test
PerfO: Performance-related outcome
PGE2: Prostaglandin E2
PRO: Patient-reported outcome
RRMS: Relapse-remitting multiple sclerosis
SF-36: Short Form Health Survey-36
soluble TNFR1: Soluble tumor necrosis factor receptors 1
SPMS: Secondary progressive multiple sclerosis
TGF-β: Transforming growth factor beta
TLR-9: Toll-like receptor 9
VCAM1: Vascular cell adhesion molecule 1
VEGF: Vascular endothelial growth factor
